# Beat-to-Beat P-Wave Analysis Outperforms Conventional P-Wave Indices in Identifying Patients with a History of Paroxysmal Atrial Fibrillation during Sinus Rhythm

**DOI:** 10.3390/diagnostics11091694

**Published:** 2021-09-17

**Authors:** Dimitrios Tachmatzidis, Dimitrios Filos, Ioanna Chouvarda, Anastasios Tsarouchas, Dimitrios Mouselimis, Constantinos Bakogiannis, Charalampos Lazaridis, Konstantinos Triantafyllou, Antonios P. Antoniadis, Nikolaos Fragakis, Georgios Efthimiadis, Nicos Maglaveras, Dimitrios G. Tsalikakis, Vassilios P. Vassilikos

**Affiliations:** 1 3rd Cardiology Department, Hippokrateion General Hospital, Aristotle University of Thessaloniki, 546 42 Thessaloniki, Greece; tasos.tsarouchas@gmail.com (A.T.); dimitriosmouselimis@gmail.com (D.M.); bakogianniscon@gmail.com (C.B.); charalamposlazaridis86@gmail.com (C.L.); kostrianta@hotmail.com (K.T.); aantoniadis@gmail.com (A.P.A.); nfrag@auth.gr (N.F.); vvassil@med.auth.gr (V.P.V.); 2 Lab of Computing, Medical Informatics and Biomedical Imaging Technologies, School of Medicine, Aristotle University of Thessaloniki, 541 24 Thessaloniki, Greece; dimitrisfilos@gmail.com (D.F.); ioanna@med.auth.gr (I.C.); nicmag@auth.gr (N.M.); 3 1st Cardiology Department, AHEPA University Hospital, Aristotle University of Thessaloniki, 546 21 Thessaloniki, Greece; geythymi@auth.gr; 4 Department of Informatics and Telecommunications Engineering, University of Western Macedonia, 501 00 Kozani, Greece; dtsalikakis@uowm.gr

**Keywords:** P-wave indices, beat to beat, P-wave analysis, atrial fibrillation, wavelet analysis, diagnostic model

## Abstract

Early identification of patients at risk for paroxysmal atrial fibrillation (PAF) is essential to attain optimal treatment and a favorable prognosis. We compared the performance of a beat-to-beat (B2B) P-wave analysis with that of standard P-wave indices (SPWIs) in identifying patients prone to PAF. To this end, 12-lead ECG and 10 min vectorcardiogram (VCG) recordings were obtained from 33 consecutive, antiarrhythmic therapy naïve patients, with a short history of low burden PAF, and from 56 age- and sex-matched individuals with no AF history. For both groups, SPWIs were calculated, while the VCG recordings were analyzed on a B2B basis, and the P-waves were classified to a primary or secondary morphology. Wavelet transform was used to further analyze P-wave signals of main morphology. Univariate analysis revealed that none of the SPWIs performed acceptably in PAF detection, while five B2B features reached an AUC above 0.7. Moreover, multivariate logistic regression analysis was used to develop two classifiers—one based on B2B analysis derived features and one using only SPWIs. The B2B classifier was found to be superior to SPWIs classifier; B2B AUC: 0.849 (0.754–0.917) vs. SPWIs AUC: 0.721 (0.613–0.813), *p* value: 0.041. Therefore, in the studied population, the proposed B2B P-wave analysis outperforms SPWIs in detecting patients with PAF while in sinus rhythm. This can be used in further clinical trials regarding the prognosis of such patients.

## 1. Introduction

Atrial fibrillation (AF)—the most common sustained cardiac arrhythmia—while not a life-threatening condition itself, leads to an increased risk of stroke or heart failure and high rates of mortality [1]. Therefore, early detection and diagnosis of AF is a critical issue for all health stakeholders. Identifying individuals at a higher risk of developing AF is feasible using predictive models based on clinical variables, circulating biomarkers, and P-wave indices (PWIs) [2].

The P-wave on the resting 12-lead electrocardiogram (ECG) is a representation of atrial depolarization, providing a wealth of information valuable in predicting incident AF. The PR interval and other standard PWIs (SPWIs) such as P-wave duration, P-wave terminal force in lead V_1_ (PTFV_1_), interatrial block (IAB), P-wave area, and P-wave axis, along with QT interval, ECG-derived ventricular hypertrophy, and incidence of atrial and ventricular ectopy have been proposed as potential identifiers of individuals at risk for developing AF [3]. Similarly, clinical risk scores such as the CHA_2_DS_2_-VASc score have been directly associated with the incidence of new-onset AF [4]. Furthermore, the association of PWIs with conditions beyond AF underlines their clinical importance. Therefore, PTFV_1_, P-wave duration, advanced IAB (a-IAB), and P-wave area have been correlated with stroke incidence [5,6]. Moreover, the addition of the P-wave axis to the standard CHA_2_DS_2_-VASc score improved its performance in stroke prediction [7].

More sophisticated ECG indicators derived from P-wave wavelet analysis have been used in patients with no evidence of structural heart disease or any other predisposing factors to successfully identify those at risk of developing AF [8]. It has also been shown that during sinus rhythm, multiple P-wave morphologies are present more frequently in patients with a history of AF [9], indicating differences in the electrical substrate of the atria [10]. Thus, morphological variability, along with beat-to-beat (B2B) wavelet analysis, is proposed as an integrated approach to P-wave analysis.

The present study aims to compare the performance of B2B P-wave analysis with that of traditional predictors of paroxysmal atrial fibrillation (PAF), such as SPWIs, in identifying patients with a history of PAF during sinus rhythm. The superiority of a B2B-P-wave analysis-based classifier over a SPWIs-based one would support the hypothesis that P-wave B2B morphological alterations provide a wealth of information on atrial predisposition to AF that SPWIs cannot reveal.

## 2. Materials and Methods 

We invited 40 consecutive patients with newly diagnosed (less than a month) PAF and no history of antiarrhythmic medication to participate in the study; 60 age- and gender-matched individuals without any history of PAF or structural heart disease were used as the control group. Patients with comorbidities, such as previous cardiovascular surgery, previous cardiac ablation, heart failure NYHA class III-IV, severe valvular heart disease, prosthetic valves, reduced life expectancy, age > 75 years, atrioventricular block, presence of implanted pacemaker or cardiac defibrillator, moderate/severe renal or hepatic impairment, were excluded from the analysis. A complete medical history was obtained from all study volunteers, and both groups underwent clinical examination and calculation of the CHA_2_DS_2_-VASc score. Eventually, 33 patients with a PAF history and 56 controls fulfilled inclusion criteria. The baseline characteristics of the two groups can be found in Table 1.

Standard 12-lead ECGs were obtained from all participants. The recordings were performed at least 7 days apart from any AF episode to minimize the potential effect of myocardial stunning. The ECGs were scanned, stored in digital format, magnified sufficiently, and analyzed with digital image processing software (imagej.nih.gov/ij, accessed on 21 October 2020). Additionally, three-orthogonal axis system (X-frontal, Y-vertical, and Z-sagittal axis) vectorcardiographic (VCG) signals of 10 min duration were also recorded at the same time, with study individuals resting in the supine position, using a high sampling rate (1000 Hz) Galix GBI-3S Holter monitor.

All participants were informed about the scope of the study and gave written informed consent. The study complied with the Declaration of Helsinki and was approved by the Special Purpose General Assembly of Aristotle University School of Medicine (#8, approved on 9 September 2016).

### 2.1. Standard P-Wave Indices

#### 2.1.1. The 12-Lead ECG Indices

Eight conventional 12 lead ECG PWIs, PR duration, P-wave duration, P-wave dispersion, P-wave peak time, P-wave axis, P-wave voltage in lead I, PTFV_1_, and P-wave area were measured by three observers, and mean values were calculated. P-wave dispersion was defined as the difference between the longest, and the shortest P-wave duration measured in any of the standard ECG leads. P-wave peak time is a novel index, equal to the duration between the beginning and peak of the P-wave measured in leads II or V_1_ [11]. An abnormal P-wave axis was determined as a frontal P-wave axis less than 0° or more than 75° [3], while PTFV_1_ was calculated as the amplitude-duration product of the terminal negative component of the P-wave in lead V_1_ [12]. P-wave area (mV × ms) was measured as the sum of the absolute areas underneath the positive and negative P-wave deflections, and the maximum area from among the 12 leads was selected [3].

Furthermore, P-wave biphasicity in inferior leads was assessed to identify IAB type [13]. Partial-IAB (p-IAB) was defined as a P-wave ≥ 120 ms without a negative deflection in the inferior leads (II, III, aVF), and advanced IAB (a-IAB) as a P-wave ≥ 120 ms, along with biphasic morphology in inferior leads. Finally, a relatively new composite score, the MVP score (morphology–voltage–P-wave duration), was calculated, assigning up to two points to each of the three components [14] (Appendix A.1.1, Appendix A.1.2, Appendix A.1.3, Appendix A.1.4, Appendix A.1.5, Appendix A.1.6, Appendix A.1.7).

#### 2.1.2. Orthogonal Morphology

Orthogonal P-wave morphology was accessed according to P-wave positive/negative deflection, or biphasicity, in leads X, Y, and Z. Three predefined types (orthogonal type 1, 2, or 3) indicative of the interatrial conduction route were considered [15]. In types 1 and 2, leads X and Y are positive, and lead Z is either negative or biphasic, while in type 3 lead X is positive and lead Y is biphasic. (Appendix A.1.8).

In conclusion, a total of fourteen SPWIs was taken into consideration; the eight conventional 12 lead ECG PWIs, along with the MVP score, a-IAB, p-IAB, and orthogonal types 1–3.

### 2.2. P-Wave Beat-to-Beat Analysis

#### 2.2.1. Beat-to-Beat Classification into Main and Secondary Morphology

Orthogonal vectorcardiograms were further studied to accomplish B2B analysis. Signal processing was performed using MATLAB R2015a, The MathWorks, Inc., Natick, MA, USA. Following an automated signal pre-processing procedure, consisting of denoising and QRS complex detection, artifacts and ectopic beats were removed in a semi-automated manner. According to a methodology previously described [9], where the existence of main and secondary P-wave morphologies was proposed, a clustering technique was used to classify P-waves into distinct groups of main, secondary, or other less frequent morphologies. Specifically, a signal window of 250 ms preceding every QRS complex, named P segment (P_SEG_), was defined. Following a k-means clustering method, the optimal number of clusters, which better classify the P_SEGs_ was calculated, and every P_SEG_ was allocated to a cluster. The mean morphology of the cluster containing most P_SEGs_ was used as a template for the detection of the P-waves matching the main morphology. For the remaining P_SEGs_, following the same process once again, an additional P-wave template was extracted, to detect the P-waves allocated to the secondary morphology. The beats that did not match any of the morphologies were considered as other morphologies. Therefore, the secondary morphology was not extracted merely as the mean morphology of the P-waves not matching the main morphology, but rather as the mean values of a group of beats highly correlated and truly forming a cluster. Finally, the percentage of P-waves matching the main and the secondary morphology in each lead was calculated, as well as the percentage of P-waves following simultaneously the main morphology in all three leads (Appendix A.2.1.).

#### 2.2.2. Time-Domain Analysis

Time features regarding P-wave position related to the QRS complex were calculated. Thus, the distance between P-wave onset, offset, and maximum value, and Q- or R-points were assessed for every beat allocated to the main morphology.

#### 2.2.3. Time-Frequency Domain Analysis

The P-waves of the dominant morphology were further analyzed in a B2B aspect using continuous wavelet transform, an approach already been used for processing non-stationary signals such as ECG. The base wavelet used was the complex Morlet wavelet. According to a previous study of ours, where optimal frequency zone range was investigated, analysis was performed in three non-overlapping bands (low—L: 30–70 Hz, medium—M: 70–160 Hz, and high—H: 160–200 Hz) [9]. Frequency features such as mean, median, and maximum energy, as well as time-frequency features, such as maximum energy location regarding P-wave onset, offset, or peak, were calculated (Appendix A.2.2.).

Eventually, P-wave B2B analysis, consisting of classification into primary and secondary morphology, and time-domain and time-frequency domain analyses, in three different axes, in three individual frequency zones, and both mean value and coefficient of variation calculation of selected variables ended up to a total of 262 features (Table 2).

### 2.3. Statistical Analysis

Statistical analysis data were expressed as mean ± standard deviation for continuous variables. The Mann–Whitney *U* test or the Fisher’s exact test was used, as appropriate, for statistical testing. Univariate logistic regression analysis was performed on statistically significant features to determine variables with prognostic value on detecting patients with a history of AF, and variables were ranked according to their area under the curve (AUC). Predictors were checked for collinearity, and the features with the highest AUC among those highly correlated were selected for further study.

Following multivariate logistic regression analysis, two different classifiers were developed—one based on features derived from B2B analysis and one using SPWIs. Receiver operating characteristics (ROC) curves were delineated for these classifiers, and the AUC was calculated to estimate the predictive value of each one. AUC comparison was accomplished using DeLong’s method [16]. Statistical analysis was performed using MATLAB (R2015a) computer software, and an alpha level of <0.05 was accepted as statistically significant.

## 3. Results

The studied dataset consisted of 276 features in total; of those, 262 variables were obtained from B2B analysis and 14 from SPWIs. Five SPWIs were found significantly different between the two groups, while Β2Β analysis was represented by nineteen statistically significant features; seven of them were derived from the percentage of main or secondary morphologies and twelve from spectral and temporal analysis (Table 3).

Nine traditional PWIs did not differ significantly between the two groups: PR duration, P-wave dispersion, P-wave peak time, P-wave axis, PTFV_1_, P-wave orthogonal type, and a-IAB values were comparable in control and PAF groups. On the other hand, the widely used P-wave duration was longer, and the P-wave area, along with P-wave voltage in lead I were smaller in PAF patients, compared to the control group. Moreover, p-IAB was more common and the MVP score was higher in the PAF group.

Additionally, morphology analysis revealed significant differences between main, secondary, and secondary to the main ratio in both axis X and Y, along with the percentage of P-waves following main morphology in all leads at the same time. Furthermore, both time-domain features and frequency-derived parameters in all three axes were represented by significant variables such as coefficient of variation of P-wave peak time in axis X, mean value of maximum energy in the high-frequency band in axis Y, or mean value of maximum energy location to P-wave duration in the mid-frequency band in axis Z.

Following univariate logistic regression analysis, variables were ranked according to estimated AUC, and odds ratios (OR) were calculated. Sixteen features were significant predictors, while eight variables did not reach the level of significance or AUC lower limit above 0.5 and were excluded from further analysis (Table 4).

Collinearity test revealed a strong correlation between main, secondary, and secondary to main morphology ratio in axis X, between the coefficient of variation of the distance between P-wave onset and the Q- or R-wave in axis X, and between the MVP score and P-wave duration. Only the features with the highest AUC among those highly correlated were selected (Table 4). Thus, eight B2B features and four SPWIs were finally used in multivariable regression analysis. Eventually, two classifier groups were developed: one using SPWIs and one using B2B variables. The risk of overfitting was kept low by ensuring an event-per-variable ratio above 10, and thus, only models consisting of ≤ 3 compounds were taken into consideration [17].

All suggested models for each category were compared to each other in terms of AUC, and those with the highest performance in our data sample were selected (Table 5). In the first case, a B2B classifier consisting of the following three features was proposed: (i) coefficient of variation of distance between the P-wave peak and Q-wave in Χ axis, (ii) percentage of P-waves allocated in main morphology in X axis, and (iii) mean value of maximum energy in the high-frequency band in the Y axis. In the second case, the model with the highest AUC among all SPWIs classifiers consisted of (i) p-IAB, (ii) P-wave voltage in lead I, and (iii) P-wave duration.

**Figure 1:**
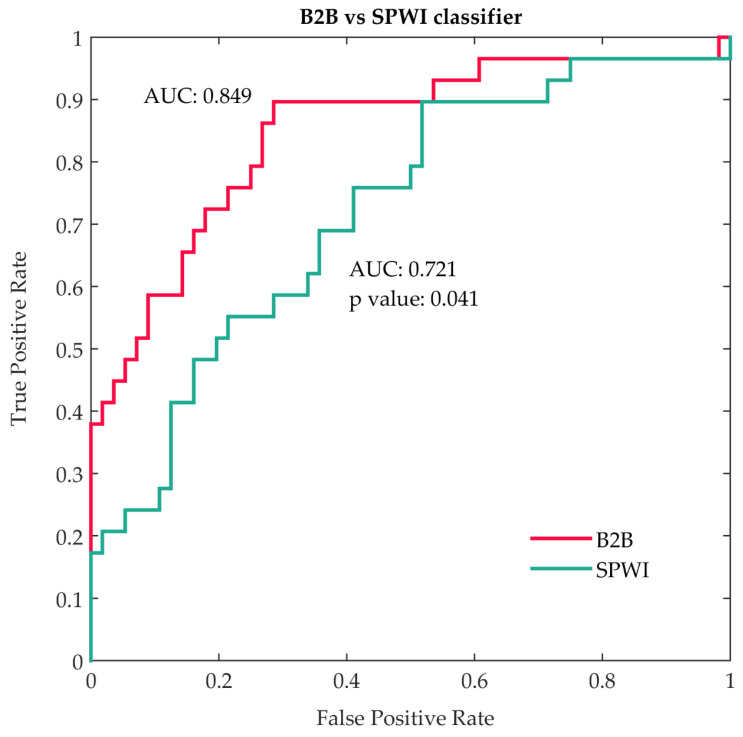
Comparison between beat-to-beat and standard P-wave indices classifiers. Abbreviations: B2B: beat to beat; SPWI: standard P-wave indices; AUC: area under the curve.

These two classifiers were finally compared to each other, and the B2B model was found superior to the SPWIs model: B2B AUC: 0.849 (0.754–0.917) vs. SPWIs AUC: 0.721 (0.613–0.813), *p* value: 0.041 (Figure 1).

## 4. Discussion

In the current study, we assessed the performance of B2B P-wave analysis in distinguishing PAF patients from individuals with no history of AF, compared to that of standard PWIs. In our sample, a B2B-derived model outperformed a classifier based on established ECG-based PAF predictors.

Univariate analysis revealed that only B2B indices, and none SPWI, achieved an acceptable AUC > 0.7 in predicting PAF (Table 4). Furthermore, the B2B classifier consists of three components coming from three different fields of B2B analysis: main and secondary morphology analysis (percentage of P-waves allocated in main morphology in X axis), temporal analysis (coefficient of variation of distance between the P-wave peak and Q-wave in Χ axis), and wavelet analysis of main P-wave morphology (mean value of maximum energy in the high-frequency band in the Y axis), underlying the importance of an integrated approach to PAF prediction. Additionally, the SPWI classifier consists of p-IAB, voltage in lead I, and P-wave duration, features that, interestingly, are also compounds of the recently developed MVP score [14].

Many PWIs have been previously found to be highly correlated to AF development. P-wave duration may be the most widely studied among them. In our study, P-wave duration along with voltage in lead I, and patrial IAB was one of the variables included in the SPWI classifier. In a meta-analysis of Framingham Heart Study (FHS) and Atherosclerosis Risk in Communities (ARIC) study, P-wave duration > 120 ms was significantly associated with AF [18]. Moreover, low P-wave amplitude in lead I has been related to displaced interatrial conduction and AF recurrence in another study [19].

Additionally, in the aforementioned work, the P-wave area did not show a robust correlation with AF, and PTFV_1_ predicted AF only in the ARIC study and not in FHS [18]. However, PTFV_1_ has been found in other studies to be significantly associated with AF occurrence [3,12] but not in ours.

Advanced interatrial block (a-IAB) is an established electrocardiographic phenotype, that has been thoroughly studied. However, in Regicor Study it was found that a-IAB did not seem to provide an additional AF risk beyond that of P-wave duration [20], which was concordant with our findings. Risk factors for developing a-IAB such as aging, hypertension, and obesity are similar to those for developing AF, interpreting the relationship between a-IAB and AF [20]. Since, patients selected in our study were relatively young, with a short history of PAF and few other comorbidities, there were very few cases of a-IAB, not enough to support a statistically significant result. On the contrary, p-IAB was found to be a quite powerful predictor and thus was included in the proposed SPWI classifier.

One of the novelties in our work is the study of P-wave variability with means of B2B analysis. Most previous studies on P-wave analysis were conducted on 10 s ECG recordings or signal-averaged ECGs. However, a B2B approach on a P-wave study is feasible and effective [21,22]. In fact, in our study, not only we managed to observe P-wave variability in 10 min recordings, but we also succeeded in depicting and quantifying primary and secondary morphologies using an algorithm formerly described and repeatedly tested for consistency in various datasets. A higher percentage of recorded beats belonging to secondary morphologies and a lower percentage of beats of dominant morphology, in various orthogonal axes, were strongly associated with PAF, both in the current dataset and in a different dataset, previously studied [9]. This P-wave variability can only be reliably quantified in relatively lengthy ECG recordings such as the ones we use in the current study and could be proved to be the main advantage of the proposed modality. In fact, since the whole process is largely automated, the workload required to analyze a 10-minute recording is considerably low. 

In addition, it is of great importance to underline that the patients who participated in our work had a noticeably short history of PAF (less than a month), were relatively young (mean age: 55.4 ± 12.6), and had a low CHA_2_DS_2_-VASc score (1.2 ± 1.3). Thus, using the proposed analysis it is feasible to detect predisposition to AF in the early stages. Similarly, impaired interatrial conduction, assessed utilizing orthogonal morphology type, has been observed in young patients without comorbidities, implying that alterations in atrial electrophysiology may be the cause rather than the consequence of AF [23]. Moreover, in our study, patients receiving antiarrhythmic drugs were excluded, so it is safe to conclude that there was no interference to results caused by antiarrhythmic medication.

The case–control design of this study is a relative limitation but considered acceptable for a study investigating a novel diagnostic modality. Larger sample size would probably provide more robust results. In this work, simple logistic regression was employed, but in the future, more sophisticated artificial intelligence-based classifiers trained with features derived from larger populations are expected to reach a higher level of accuracy.

Although a well-known ECG-derived AF predictor, signal-averaged ECG (SAECG) was not analyzed in the current study. Recording atrial late potentials is technically challenging; thus, the hypothesis that low-amplitude atrial potentials are associated with AF was initially rejected [24]. However, using improved techniques, several SAECG P-wave indices were studied. In fact, atrial SAECG is widely used to calculate filtered P-wave duration (FPD), a more precise and reproducible measure of P-wave duration. On the contrary, SAECG late potential parameters, such as root mean square voltage of terminal P-wave segments, supposed to represent the activation of the left pulmonary veins [25], have shown little reproducibility and their clinical importance is controversial [26]. Therefore, in patients with PAF, FPD was longer, compared to normal controls, while no statistical difference was found in atrial late potentials [27]. Furthermore, FDP can identify hypertensive PAF patients while in sinus rhythm [28] and, along with left atrial dimension, is considered significant predictor of transmission from paroxysmal to persistent or permanent AF [29,30].

Platonov et al. have shown that fibrosis and fatty infiltration were more manifest in autopsies of patients with permanent AF than in those with paroxysmal AF, regardless of patients’ age [31]. Moreover, P-wave duration and amplitude show a significant correlation with low-voltage area size and may be used as a non-invasive tool to predict severe scarring, as experimental and simulation studies suggest [32,33]. Therefore, PWIs probably predict AF progression by reflecting atrial fibrosis extension. Furthermore, B2B P-wave variability in patients with paroxysmal AF could be explained by variability in sinoatrial node exit location in combination with slow conducting regions, such as scars, as has been shown in computational simulation studies [34,35].

In future studies, an association between B2B indices and AF predictors other than SPWIs can be examined. Thus, a correlation between B2B analysis and echocardiographic parameters of left atrial size and function or characteristics of the left atrial substrate such as low voltage areas would be a potential research topic. Moreover, prospective studies can be conducted to determine B2B predictors’ ability to detect clinical conditions beyond AF, such as embolic strokes of an undetermined source (ESUS).

## 5. Conclusions

Early identification of patients in the absence of organic heart disease or other predisposing factors, who are prone to PAF is a crucial issue for all health stakeholders. B2B P-wave analysis is a novel, effective approach for early AF prediction and seems that it is superior to the conventional PWIs analysis. Larger datasets will be necessary to aid the development of artificial intelligence models based on this analysis, to accurately predict PAF during sinus rhythm.

## Figures and Tables

**Table 1:** Baseline characteristics of study participants.

Patient Parameters	PAF (*n* = 33)	Healthy (*n* = 56)	*p* Value
Age	55.4 ± 12.6	55.2 ± 5.7	0.933
Male sex (%)	24 (72.7)	38 (67.9)	0.812
Body mass index (kg/m^2^)	28.2 ± 4.6	26.3 ± 5.9	0.756
Hypertension (%)	14 (42.4)	12 (21.4)	0.053
Diabetes (%)	0 (0.0)	1 (1.8)	1.000
Dyslipidemia (%)	10 (30.3)	14 (25.0)	0.626
CHA_2_DS_2_-VASc score	1.2 ± 1.3	0.7 ± 0.8	0.123

Independent samples Mann–Whitney *U* test, Fisher’s exact test. Continues variables are reported mean ± SD. Categorical variables are reported *n* (%).

**Table 2:** Feature categories derived from beat-to-beat analysis.

Beat-to-Beat Analysis Features	Leads	Freq. Zones	Mean/cv	Subtotal
Percentage of P-waves matching main morphology	3	-	1	3
Percentage of P-waves matching secondary morphology	3	-	1	3
Secondary to main P-waves ratio	3	-	1	3
Percentage of P-waves matching main morphology in all axes at the same time	1	-	1	1
Distance between P-wave onset to R-wave	3	-	2	6
Distance between P-wave onset to Q-wave	3	-	2	6
Distance between P-wave peak to R-wave	3	-	2	6
Distance between P-wave peak to Q-wave	3	-	2	6
Distance between P-wave offset to R-wave	3	-	2	6
Distance between P-wave offset to Q-wave	3	-	2	6
Distance between P-wave onset to P-wave peak	3	-	2	6
Distance between P-wave onset to P-wave peak/P-wave duration	3	-	2	6
Distance between P-wave peak to P-wave offset	3	-	2	6
Distance between P-wave onset and maximum energy location	3	3	2	18
Distance between P-wave onset and maximum energy location normalized to P-wave duration	3	3	2	18
Distance between maximum energy location and P-wave offset	3	3	2	18
Distance between maximum energy location and R-wave	3	3	2	18
Distance between maximum energy location and Q-wave	3	3	2	18
Distance between P-wave peak and maximum energy location	3	3	2	18
Maximum energy value	3	3	2	18
Mean energy value	3	3	2	18
Mean energy value normalized to P-wave duration	3	3	2	18
Median energy value	3	3	2	18
Median energy value normalized to P-wave duration	3	3	2	18
Total				262

Features derived from beat-to-beat P-wave morphology and wavelet analysis. Abbreviations: leads (orthogonal leads X, Y, Z; freq. zone: frequency zones (low, mid, and high); mean: mean value; cv: coefficient of variation (SD/mean).

**Table 3:** P-wave indices and beat-to-beat variables in PAF patients and healthy controls.

Features	Healthy (*n* = 56)	AF (*n* = 33)	*p* Value
Standard P-wave indices			
P-wave area (ms × mV)	11.1 ± 4.5	8.7 ± 6.6	0.025
P-wave duration (ms)	112.2 ± 16.2	122.6 ± 19.5	0.006
Partial Interatrial Block	8 (14.3%)	14 (42.4%)	0.003
P-wave voltage in lead I (mV × 10^−3^)	145.3 ± 50.5	110.4 ± 56.1	0.005
MVP ECG score	2.036 ± 1.436	3.000 ± 1.561	0.005
PR duration (ms)	182.6 ± 18.8	183.9 ± 27.5	0.763
P-wave dispersion (ms)	25.1 ± 14.5	24.2 ± 13.2	0.799
P-wave peak time	67.9 ± 9.7	69.1 ± 20.6	0.349
P-wave axis	47.1 ± 31.5	53.6 ± 22.3	0.075
P-wave Terminal Force in lead V_1_ (ms × mV)	5.1 ± 4.5	5.2 ± 5.5	0.555
P-wave Terminal Force in lead V_1_ > 4 ms × mV	18 (32.1%)	10 (30.3%)	0.857
Advanced Interatrial Block	11 (19.6%)	6 (18.2%)	0.866
Orthogonal type 1	14 (25.0%)	6 (18.2%)	0.457
Orthogonal type 2	39 (69.6%)	22 (66.7%)	0.770
Orthogonal type 3	3 (5.4%)	2 (6.1%)	0.889
Beat-to-beat analysis			
Morphologies (%)			
P-waves matching main morphology–X	99.7 ± 0.8	96.6 ± 6.4	<0.001
P-waves matching main morphology–Y	99.0 ± 3.8	95.8 ± 8.0	<0.001
P-waves matching secondary morphology–X	0.2 ± 0.6	1.5 ± 3.1	<0.001
P-waves matching secondary morphology–Y	0.4 ± 1.4	2.0 ± 4.6	0.017
P-waves matching main morphology–all leads simultaneously	95.2 ± 7.7	89.1 ± 12.3	0.041
Secondary to main P-waves ratio–X	0.002 ± 0.006	0.02 ± 0.04	<0.001
Secondary to main P-waves ratio–Y	0.005 ± 0.02	0.03 ± 0.07	0.016
Time domain (ms)			
P-wave peak to R-wave–X, cv	0.08 ± 0.2	0.1 ± 0.08	<0.001
P-wave peak to Q-wave–X, cv	0.03 ± 0.4	0.2 ± 0.2	<0.001
P-wave onset to R-wave–X, cv	0.03 ± 0.02	0.05 ± 0.04	0.004
P-wave onset to Q-wave–X, cv	0.04 ± 0.02	0.08 ± 0.06	0.004
P-wave onset to P-wave peak (peak time)–X, cv	0.05 ± 0.03	0.08 ± 0.06	<0.001
P-wave peak to P-wave offset–X, cv	0.1 ± 0.5	0.09 ± 0.06	<0.001
P-wave peak to P-wave offset–Y, cv	0.05 ± 0.03	0.1 ± 0.2	0.041
Time-frequency domain (ms)			
Maximum energy location to P-wave offset–mid band, Z, mean	61.1 ± 18.6	53.8 ± 20.1	0.044
Maximum energy location to P-wave onset, normalized to P-wave duration–high band, Z, mean	0.6 ± 0.1	0.6 ± 0.09	0.049
Maximum energy location to P-wave onset, normalized to P-wave duration–mid band, Z, mean	0.6 ± 0.1	0.6 ± 0.1	0.034
Frequency domain (μV^2^)			
Maximum energy–high band, Y, mean	0.2 ± 0.1	0.1 ± 0.09	0.003
Maximum energy–high band, Y, cv	0.5 ± 0.1	0.4 ± 0.2	0.028

Continuous variables are reported as mean ± SD. Categorical variables are reported as *n* (%). In beat-to-beat analysis, only variables with a significant value of *p* < 0.05 are reported. Abbreviations: cv: coefficient of variation; X, Y, Z: orthogonal leads X, Y, Z.

**Table 4:** Univariate regression analysis of potential prognostic factors on the presence of atrial fibrillation.

Rank	Feature	Type	AUC	AUC Limits	OR	95% CI	*p* Value
1	Peak time–X, cv	B2B	0.726	0.606–0.846	2.672	1.463–4.880	**0.001**
2	P-wave peak to Q-wave–X, cv	B2B	0.725	0.614–0.836	33.513	3.904–287.692	**0.001**
3	Main morphology %–Y	B2B	0.723	0.616–0.830	0.553	0.307–0.996	**0.048**
4	P-wave peak to R-wave–X, cv	B2B	0.716	0.598–0.834	1.130	0.733–1.742	0.581
5	P-wave peak to P-wave offset–X, cv	B2B	0.710	0.590–0.830	0.935	0.574–1.523	0.788
6	Main morphology %–X	B2B	0.698	0.582–0.813	0.081	0.015–0.434	**0.003**
7	Max energy–high band, Y, mean	B2B	0.681	0.562–0.799	0.550	0.326–0.930	**0.026**
8	P-wave onset to Q-wave–X, cv	B2B	0.679	0.562–0.796	2.542	1.421–4.547	**0.002**
9	P-wave voltage in lead I	SPWI	0.679	0.558–0.800	0.493	0.300–0.810	**0.005**
10	* Secondary to main morphology ratio %–X	B2B	0.679	0.586–0.771	7.525	1.638–34.562	**0.009**
11	* Secondary morphology %–X	B2B	0.678	0.585–0.771	4.897	1.503–15.959	**0.008**
12	* P-wave onset to R-wave–X, cv	B2B	0.676	0.556–0.796	2.399	1.331–4.324	**0.004**
13	P-wave duration	SPWI	0.675	0.555–0.795	1.842	1.150–2.950	**0.011**
14	* MVP score	SPWI	0.673	0.560–0.787	1.921	1.209–3.052	**0.006**
15	P-wave area	SPWI	0.653	0.528–0.779	0.587	0.357–0.964	**0.035**
16	Partial Interatrial Block	SPWI	0.641	0.543–0.738	1.906	1.225–2.964	**0.004**
17	Max energy–high band–Y, cv	B2B	0.640	0.510–0.770	0.704	0.438–1.130	0.146
18	Max energy location to Pdur.–mid band, Z, mean	B2B	0.633	0.510–0.756	1.680	1.024–2.755	**0.040**
19	Main morphology %–all axes	B2B	0.631	0.500–0.761	0.527	0.326–0.851	**0.009**
20	Max energy location to P offset–mid band, Z, mean	B2B	0.630	0.506–0.755	0.668	0.420–1.062	0.088
21	Max energy location to Pdur.–high band, Z, mean	B2B	0.624	0.502–0.746	1.575	0.973–2.551	0.065
22	P-wave peak to P-wave offset time–Y, cv	B2B	0.620	0.496–0.743	6.590	0.953–45.565	0.056
23	Secondary to main morphology ratio %–Y	B2B	0.600	0.505–0.695	1.933	0.868–4.306	0.107
24	Secondary morphology %–Y	B2B	0.600	0.504–0.695	1.830	0.928–3.611	0.081

Features ranking according to AUC. Bold values denote statistical significance at the *p* < 0.05 level. Features marked with an asterisk (*) were excluded from further analysis due to collinearity. Abbreviations: X, Y, Z: orthogonal leads X, Y, Z; Pdur.: P-wave duration; mean: mean value; cv: coefficient of variation; B2B: beat-to-beat feature; SPWI: standard P-wave index; AUC: area under the curve; OR: odds ratio; CI: confidence interval.

**Table 5:** Multivariate analysis characteristics of logistic regression-based classifiers.

Classifier	Variables	OR	95% CI	*p* Value
Beat-to-beat analysis	P-wave peak to Q-wave–X, cv	1.541	0.993–2.394	0.054
	Main morphology %–X	0.786	0.624–0.991	0.042
	Max energy–high frequency band, Y, mean	0.443	0.187–1.048	0.064
Standard P-wave indices	Partial Interatrial Block	1.979	0.550–7.122	0.296
	Voltage in lead I	0.435	0.203–0.931	0.032
	P-wave duration	1.736	0.828–3.640	0.144

Abbreviations: X, Y: orthogonal leads X, Y; cv: coefficient of variation; OR: odds ratio; CI: confidence interval.

**Figure A1:**
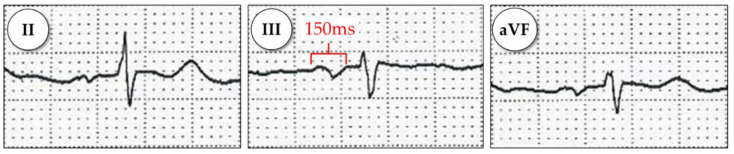
Advanced interatrial block. P-wave duration more than 120 ms along with biphasic P-wave in inferior leads II, III and aVF.

**Figure A2:**
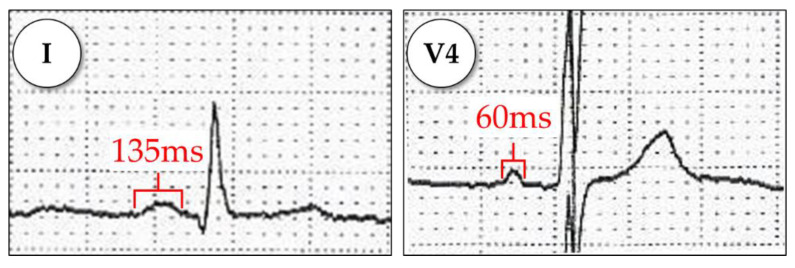
P-wave dispersion. In this case, P-wave dispersion = 75 ms (135–60 ms).

**Figure A3:**
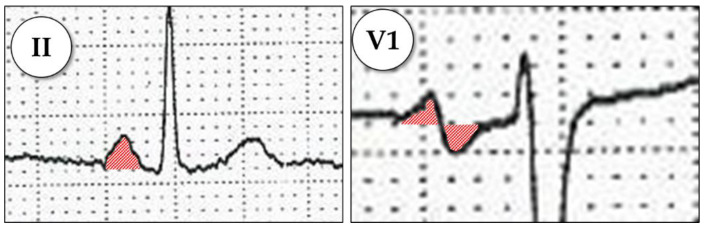
P-wave area depiction in lead II (monophasic) and lead V_1_ (biphasic).

**Figure A4:**
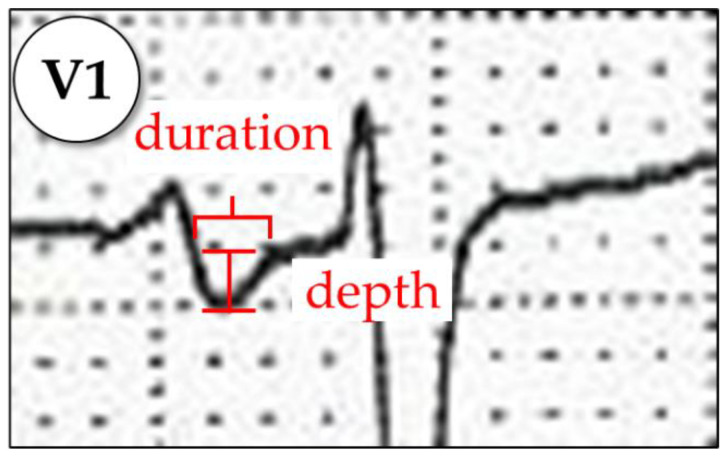
P-wave Terminal Force in V1 equals the product of negative deflection duration × depth.

**Figure A5:**
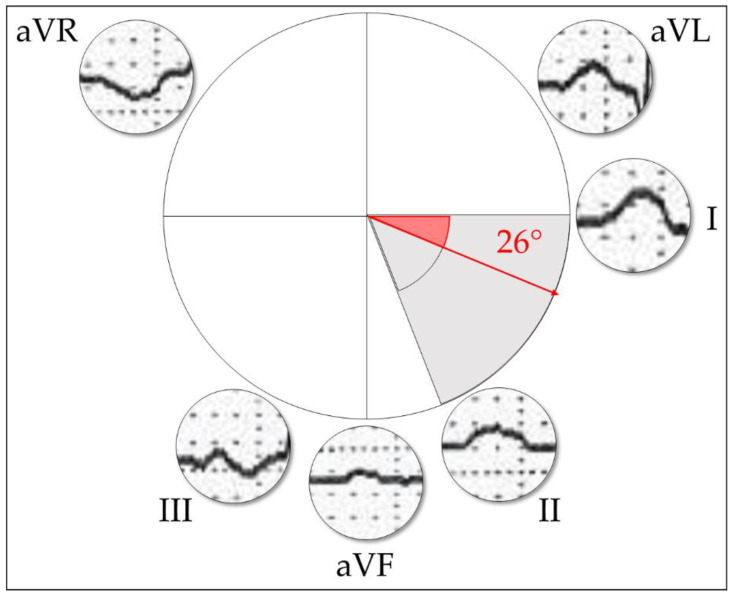
P-wave axis in the frontal plane.

**Figure A6:**
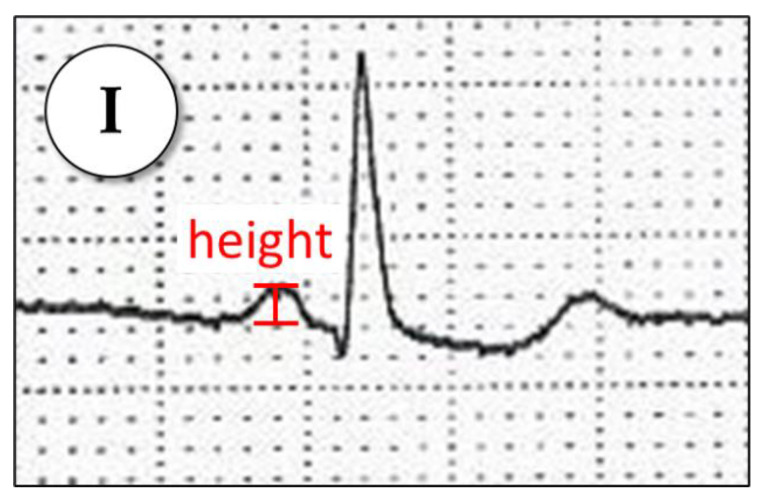
P-wave voltage in lead I.

**Figure A7:**
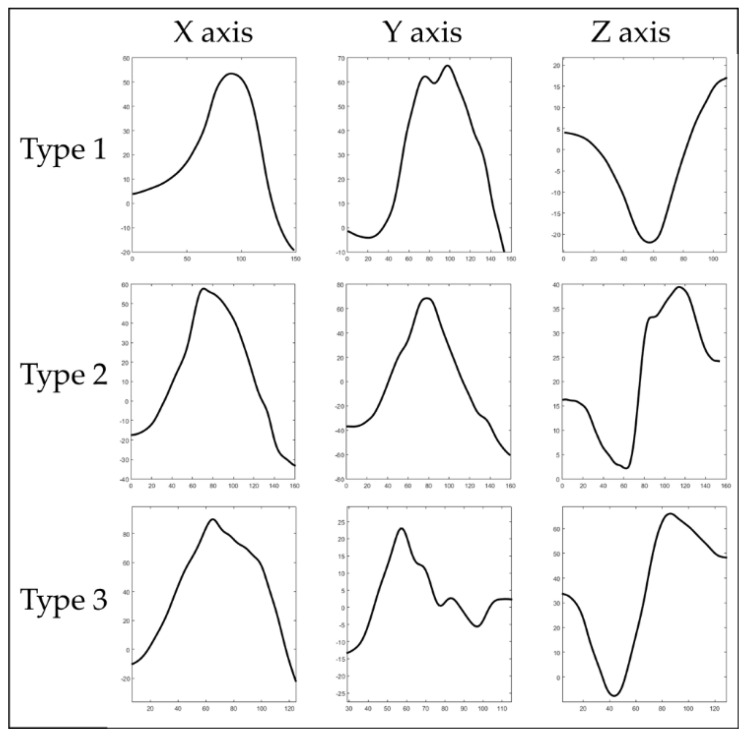
Orthogonal types 1, 2, and 3.

**Figure A8:**
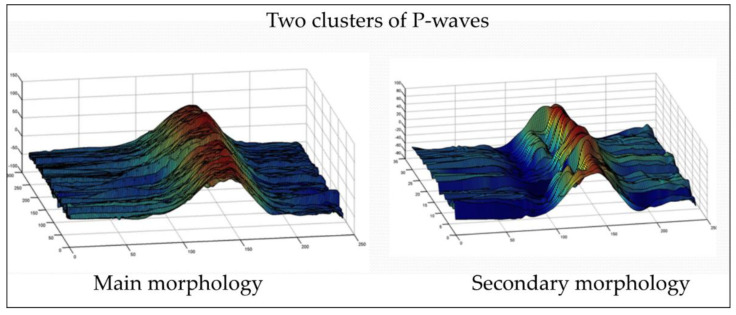
Beat-to-beat allocation of P-waves to main or secondary morphology.

**Figure A9:**
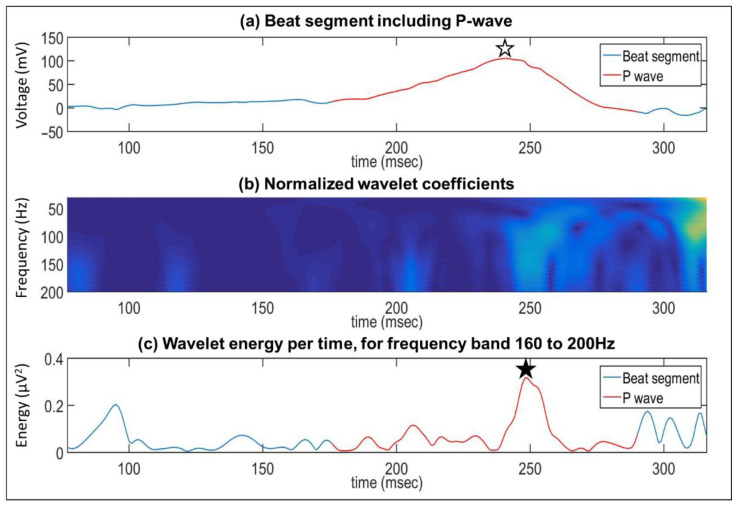
Example of wavelet analysis of an ECG signal: (**a**) ECG signal window of 250 ms preceding QRS complex. P-wave is depicted in red; (**b**) spectrogram of the same signal; (**c**) wavelet energy per time, for the high-frequency band. The white asterisk in panel (**a**) denotes the P-wave peak, while the black asterisk in panel (**c**) indicates maximum energy for this frequency zone.

## Data Availability

The data presented in this study are available on request from the corresponding author.

## Appendix A

### Appendix A.1. Standard P-Wave Indices

#### Appendix A.1.1. Advanced Interatrial Block

The advanced interatrial block is defined as P-wave duration > 120 ms along with biphasic P-wave in inferior leads (Figure A1) [13].

#### Appendix A.1.2. P-Wave Dispersion

P-wave dispersion is defined as the difference between the longest and the shortest P-wave duration measured in any of the standard ECG leads (Figure A2). P-wave dispersion is expected to be increased in PAF.

#### Appendix A.1.3. P-Wave Area

P-wave area is calculated as the sum of the absolute areas underneath the positive and negative P-wave deflections, and the maximum area from among the 12 leads is selected (Figure A3). It is decreased in PAF, with a cut-off value of 4 mV × ms [3].

#### Appendix A.1.4. P-Wave Terminal Force in V1 (PTFV1)

PTFV_1_ is calculated by multiplying the depth by the duration of the P-wave terminal negativity in V_1_ (Figure A4). A product of more than 4 mV × ms is considered abnormal [12].

#### Appendix A.1.5. P-Wave Axis

The P-wave axis is calculated in the frontal plane (Figure A5). Axis values between 0° and +75° are considered to be normal [3].

#### Appendix A.1.6. P-Wave Voltage in Lead I

P-wave voltage below 0.1 mV in lead I is considered abnormal (Figure A6) [19].

#### Appendix A.1.7. MVP Score

The MVP score (Morphology–Voltage–P-wave duration) is calculated by assigning up to two points to each one of the three components (Table A1) [14].

**Table A1:** MVP score calculation.

**Variable**	**Value**	**Score**
Morphology in inferior leads	Nonbiphasic (<120 ms)	0
	Nonbiphasic (≥120 ms)	1
	Biphasic	2
Voltage in lead I	>0.2 mV	0
	0.10–0.20 mV	1
	<0.10 mV	2
P-wave duration	<120 ms	0
	120–140 ms	1
	>140 ms	2

0–2: low probability, 3–4: intermediate probability, and 5–6: high probability of AF.

#### Appendix A.1.8. Orthogonal Types 1, 2, or 3

In orthogonal types 1 and 2, leads X and Y are positive, and lead Z is either negative or biphasic. In type 3, lead X is positive, and lead Y is biphasic (Figure A7). Each type represents a different interatrial activation route, with type 3 being highly correlated to AF [15].

### Appendix A.2. Beat-to-Beat Analysis

#### Appendix A.2.1. Main and Secondary Morphologies

Following a clustering procedure, each P-wave is allocated to main, secondary, or other less common morphologies (Figure A8). In people suffering from AF, the percentage of P-waves allocated in main morphology is smaller than in healthy individuals. On the contrary, P-waves of secondary morphology are more common in this population [9].

#### Appendix A.2.2. Wavelet Analysis

P-waves of dominant morphology are analyzed on a beat-to-beat basis, and frequency features, such as mean or maximum energy, as well as time-frequency features, such as maximum energy location regarding P-wave onset, offset, or peak are calculated (Figure A9) [8,9].

## References

[1] Dilaveris P.E., Kennedy H.L. (2017). Silent atrial fibrillation: Epidemiology, diagnosis, and clinical impact. Clin. Cardiol..

[2] Alonso A., Norby F.L. (2016). Predicting Atrial Fibrillation and Its Complications. Circ. J..

[3] German D.M., Kabir M.M., Dewland T.A., Henrikson C.A., Tereshchenko L. (2016). Atrial Fibrillation Predictors: Importance of the Electrocardiogram. Ann. Noninvasive Electrocardiol..

[4] Saliba W., Gronich N., Barnett-Griness O., Rennert G. (2016). Usefulness of CHADS_2_ and CHA_2_DS_2_-VASc Scores in the Prediction of New-Onset Atrial Fibrillation: A Population-Based Study. Am. J. Med..

[5] He J., Tse G., Korantzopoulos P., Letsas K.P., Ali-Hasan-Al-Saegh S., Kamel H., Li G., Lip G.Y., Liu T. (2017). P-Wave Indices and Risk of Ischemic Stroke. Stroke.

[6] Martínez-Sellés M., Elosua R., Ibarrola M., De Andrés M., Díez-Villanueva P., Bayés-Genis A., Baranchuk A., Bayés-De-Luna A. (2020). Advanced interatrial block and P-wave duration are associated with atrial fibrillation and stroke in older adults with heart disease: The BAYES registry. Europace.

[7] Maheshwari A., Norby F., Roetker N.S., Soliman E.Z., Koene R.J., Rooney M.R., O’Neal W.T., Shah A.M., Claggett B.L., Solomon S.D. (2019). Refining Prediction of Atrial Fibrillation-Related Stroke Using the P_2_-CHA_2_DS_2_-VASc Score: ARIC and MESA. Circulation.

[8] Dakos G., Chatzizisis Y.S., Konstantinou D., Chouvarda I., Filos D., Paraskevaidis S., Mantziari L., Maglaveras N., Karvounis H., Styliadis I. (2014). Wavelet-based analysis of P waves identifies patients with lone atrial fibrillation: A cross-sectional pilot study. Int. J. Cardiol..

[9] Filos D., Chouvarda I., Tachmatzidis D., Vassilikos V., Maglaveras N. (2017). Beat-to-beat P-wave morphology as a predictor of paroxysmal atrial fibrillation. Comput. Methods Programs Biomed..

[10] Filos D., Tachmatzidis D., Maglaveras N., Vassilikos V., Chouvarda I. (2019). Understanding the Beat-to-Beat Variations of P-Waves Morphologies in AF Patients During Sinus Rhythm: A Scoping Review of the Atrial Simulation Studies. Front. Physiol..

[11] Yıldırım E., Günay N., Bayam E., Keskin M., Ozturkeri B., Selcuk M. (2019). Relationship between paroxysmal atrial fibrillation and a novel electrocardiographic parameter P wave peak time. J. Electrocardiol..

[12] Huang Z., Zheng Z., Wu B., Tang L., Xie X., Dong R., Luo Y., Li S., Zhu J., Liu J. (2020). Predictive value of P wave terminal force in lead V1 for atrial fibrillation: A meta-analysis. Ann. Noninvasive Electrocardiol..

[13] Bayés de Luna A., Platonov P., Cosio F.G., Cygankiewicz I., Pastore C.A., Baranowski R., Bayés-Genis A., Guindo J., Viñolas X., Garcia-Niebla J. (2012). Interatrial blocks. A separate entity from left atrial enlargement: A consensus report. J. Electrocardiol..

[14] Alexander B., Milden J., Hazim B., Haseeb S., Bayes-Genis A., Elosua R., Martínez-Sellés M., Yeung C., Ma W.H., Baranchuk A. (2019). New electrocardiographic score for the prediction of atrial fibrillation: The MVP ECG risk score (morphology-voltage-P-wave duration). Ann. Noninvasive Electrocardiol..

[15] Eranti A., Carlson J., Kenttä T., Holmqvist F., Holkeri A., Haukilahti M.A., Kerola T., Aro A.L., Rissanen H., Noponen K. (2020). Orthogonal P-wave morphology, conventional P-wave indices, and the risk of atrial fibrillation in the general population using data from the Finnish Hospital Discharge Register. Europace.

[16] Delong E.R., Delong D.M., Clarke-Pearson D.L. (1988). Comparing the Areas under Two or More Correlated Receiver Operating Characteristic Curves: A Nonparametric Approach. Biometrics.

[17] Peduzzi P., Concato J., Kemper E., Holford T.R., Feinstein A.R. (1996). A simulation study of the number of events per variable in logistic regression analysis. J. Clin. Epidemiol..

[18] Magnani J.W., Zhu L., Lopez F., Pencina M.J., Agarwal S.K., Soliman E.Z., Benjamin E., Alonso A. (2015). P-wave indices and atrial fibrillation: Cross-cohort assessments from the Framingham Heart Study (FHS) and Atherosclerosis Risk in Communities (ARIC) study. Am. Heart J..

[19] Park J.K., Park J., Uhm J.S., Joung B., Lee M.H., Pak H.N. (2016). Low P-Wave Amplitude (<0.1 MV) in Lead I Is Associated with Displaced Inter-Atrial Conduction and Clinical Recurrence of Paroxysmal Atrial Fibrillation after Radiofrequency Catheter Ablation. Europace.

[20] Massó-van Roessel A., Escobar-Robledo L.A., Dégano I., Grau M., Sala J., Ramos R., Marrugat J., Bayés de Luna A., Elosua R. (2017). Analysis of the Association Between Electrocardiographic P-Wave Characteristics and Atrial Fibrillation in the REGICOR Study. Rev. Esp. Cardiol..

[21] Conte G., Luca A., Yazdani S., Caputo M.L., Regoli F., Moccetti T., Kappenberger L., Vesin J.-M., Auricchio A. (2017). Usefulness of P-Wave Duration and Morphologic Variability to Identify Patients Prone to Paroxysmal Atrial Fibrillation. Am. J. Cardiol..

[22] Censi F., Corazza I., Reggiani E., Calcagnini G., Mattei E., Triventi M., Boriani G. (2016). P-wave Variability and Atrial Fibrillation. Sci. Rep..

[23] Holmqvist F., Olesen M.S., Tveit A., Enger S., Tapanainen J., Jurkko R., Havmöller R., Haunsø S., Carlson J., Svendsen J.H. (2011). Abnormal atrial activation in young patients with lone atrial fibrillation. Europace.

[24] Engel T.R., Vallone N., Windle J. (1988). Signal-averaged electrocardiograms in patients with atrial fibrillation or flutter. Am. Heart J..

[25] Okumura Y., Watanabe I., Ohkubo K., Ashino S., Kofune M., Hashimoto K., Shindo A., Sugimura H., Nakai T., Kasamaki Y. (2007). Prediction of the Efficacy of Pulmonary Vein Isolation for the Treatment of Atrial Fibrillation by the Signal-Averaged P-Wave Duration. PACE—Pacing Clin. Electrophysiol..

[26] Savelieva I., Aytemir K., Hnatkova K., Camm A.J., Malik M. (2000). Short-, mid-, and long-term reproducibility of the atrial signal-averaged electrocardiogram in healthy subjects: Comparison with the conventional ventricular signal-averaged electrocardiogram. PACE—Pacing Clin. Electrophysiol..

[27] Dhala A., Underwood D., Leman R., Madu E., Baugh D., Ozawa Y., Kasamaki Y., Xue Q., Reddy S. (2002). Signal-Averaged P-Wave Analysis of Normal Controls and Patients with Paroxysmal Atrial Fibrillation: A Study in Gender Differences, Age Dependence, and Reproducibility. Clin. Cardiol..

[28] Aytemir K., Amasyali B., Abali G., Kose S., Kilic A., Onalan O., Tokgozoglu L., Kabakci G., Ozkutlu H., Nazli N. (2005). The signal-averaged P-wave duration is longer in hypertensive patients with history of paroxysmal atrial fibrillation as compared to those without. Int. J. Cardiol..

[29] Koide Y., Yotsukura M., Sakata K., Yoshino H., Ishikawa K. (2002). Investigation of the predictors of transition to persistent atrial fibrillation in patients with paroxysmal atrial fibrillation. Clin. Cardiol..

[30] Budeus M., Felix O., Hennersdorf M., Wieneke H., Erbel R., Sack S. (2007). Prediction of Conversion from Paroxysmal to Permanent Atrial Fibrillation. PACE—Pacing Clin. Electrophysiol..

[31] Platonov P., Mitrofanova L.B., Orshanskaya V., Ho S.Y. (2011). Structural Abnormalities in Atrial Walls Are Associated With Presence and Persistency of Atrial Fibrillation But Not With Age. J. Am. Coll. Cardiol..

[32] Schreiber T., Kähler N., Tscholl V., Nagel P., Blaschke F., Landmesser U., Attanasio P., Huemer M. (2019). Correlation of P-wave properties with the size of left atrial low voltage areas in patients with atrial fibrillation. J. Electrocardiol..

[33] Nagel C., Luongo G., Azzolin L., Schuler S., Dössel O., Loewe A. (2021). Non-Invasive and Quantitative Estimation of Left Atrial Fibrosis Based on P Waves of the 12-Lead ECG—A Large-Scale Computational Study Covering Anatomical Variability. J. Clin. Med..

[34] Filos D., Korosoglou P., Tachmatzidis D., Maglaveras N., Vassilikos V., Chouvarda I. (2018). Multiple P-Wave Morphologies in Paroxysmal Atrial Fibrillation Patients During Sinus Rhythm: A Simulation Study. Proceedings of the 2018 Computing in Cardiology Conference (Cinc).

[35] Pezzuto S., Gharaviri A., Schotten U., Potse M., Conte G., Caputo M.L., Regoli F., Krause R., Auricchio A. (2018). Beat-to-beat P-wave morphological variability in patients with paroxysmal atrial fibrillation: Anin silicostudy. Europace.

